# Supplementary figures and images for: Role of echocardiography in screening for portopulmonary hypertension in liver transplant candidates: a meta-analysis
Source: PeerJ. 2020 May 27;8:e9243. doi: 10.7717/peerj.9243 (PMC7261122; doi:10.7717/peerj.9243)

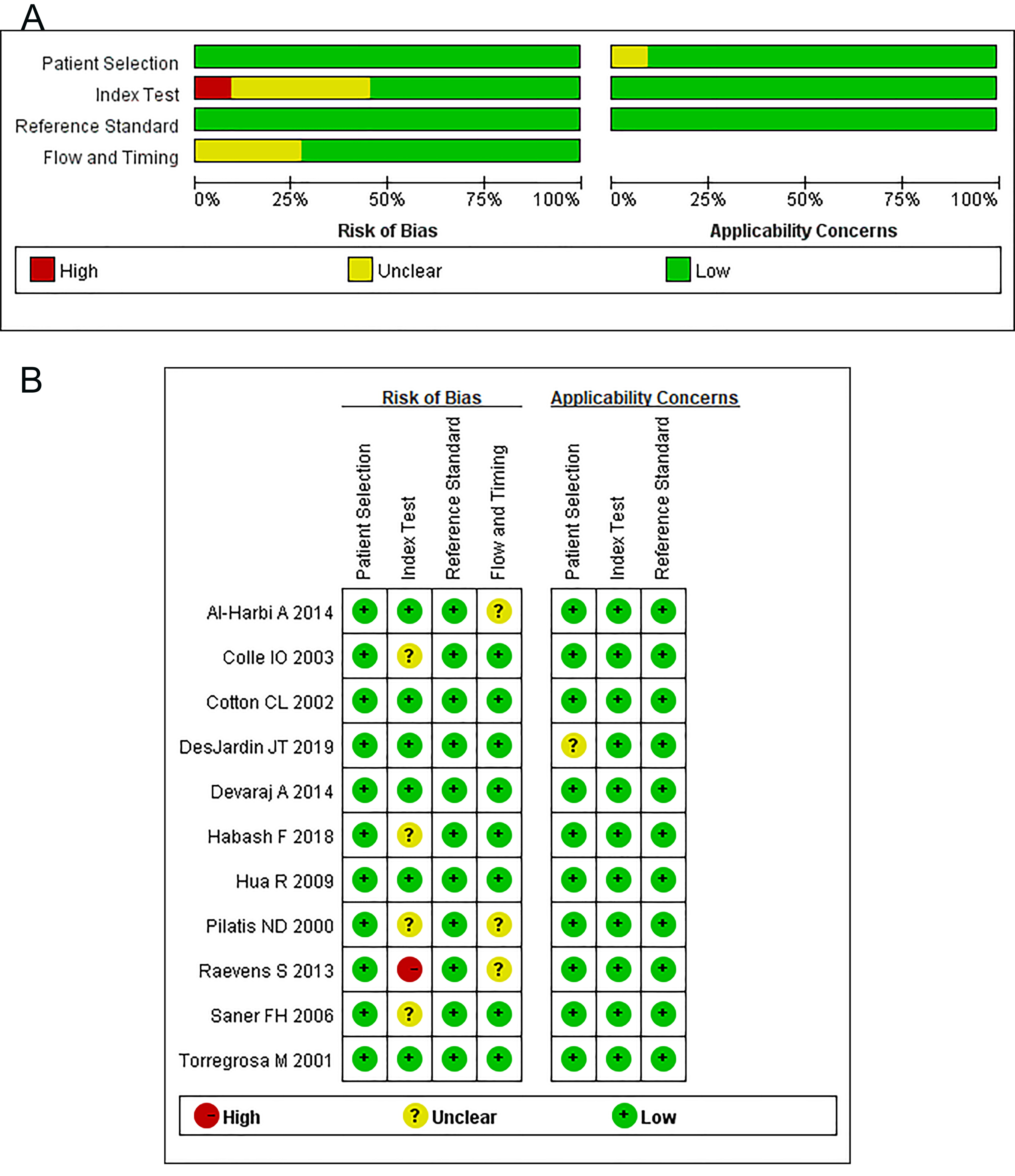

Supplement: Figure S1 — (A) Risk of bias and applicability concerns summary. (B) Risk of bias and applicability concerns graph. +, low risk; -, high risk; ?, unclear. [file peerj-08-9243-s001.png]

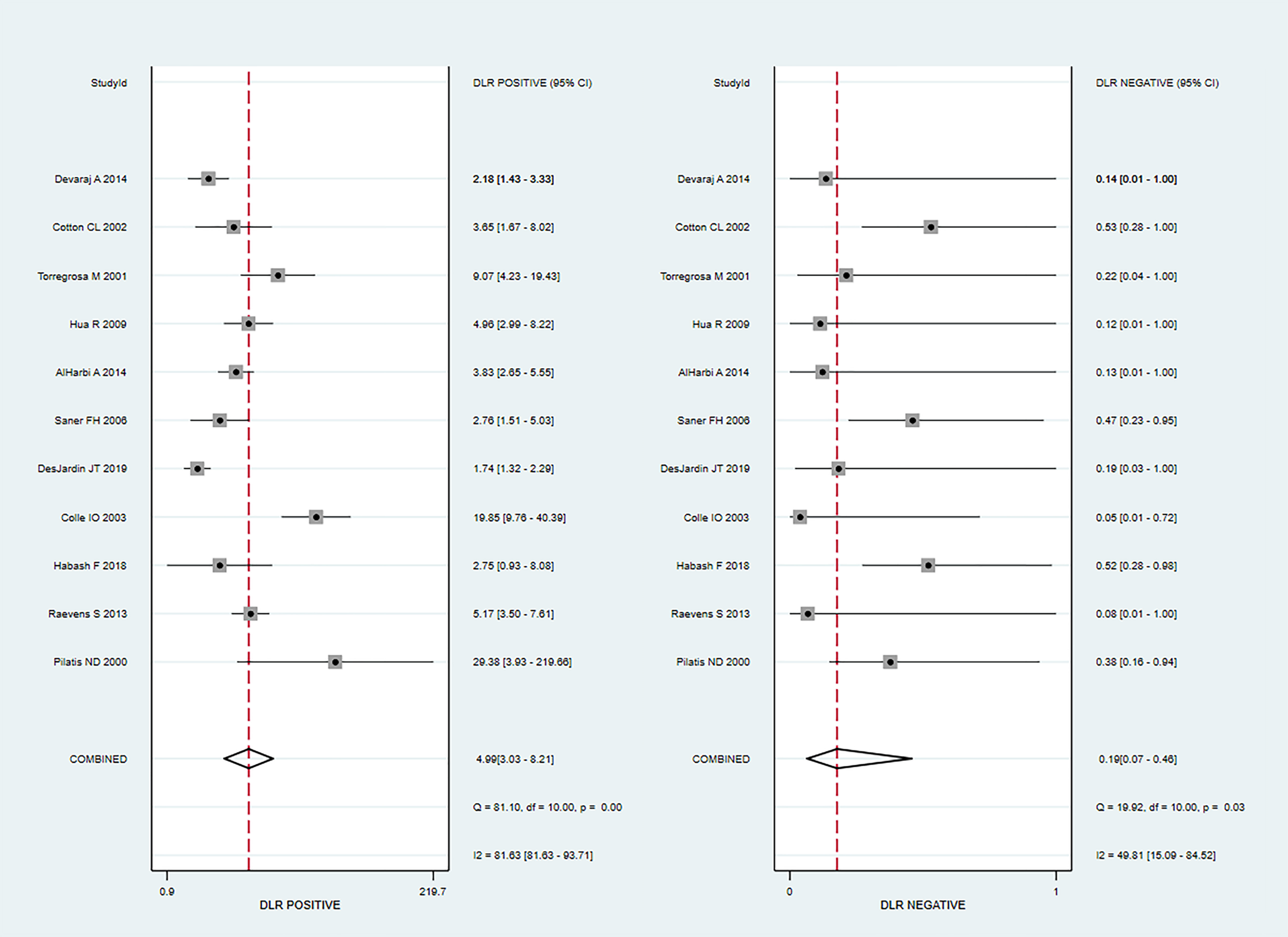

Supplement: Figure S2 [file peerj-08-9243-s002.png]

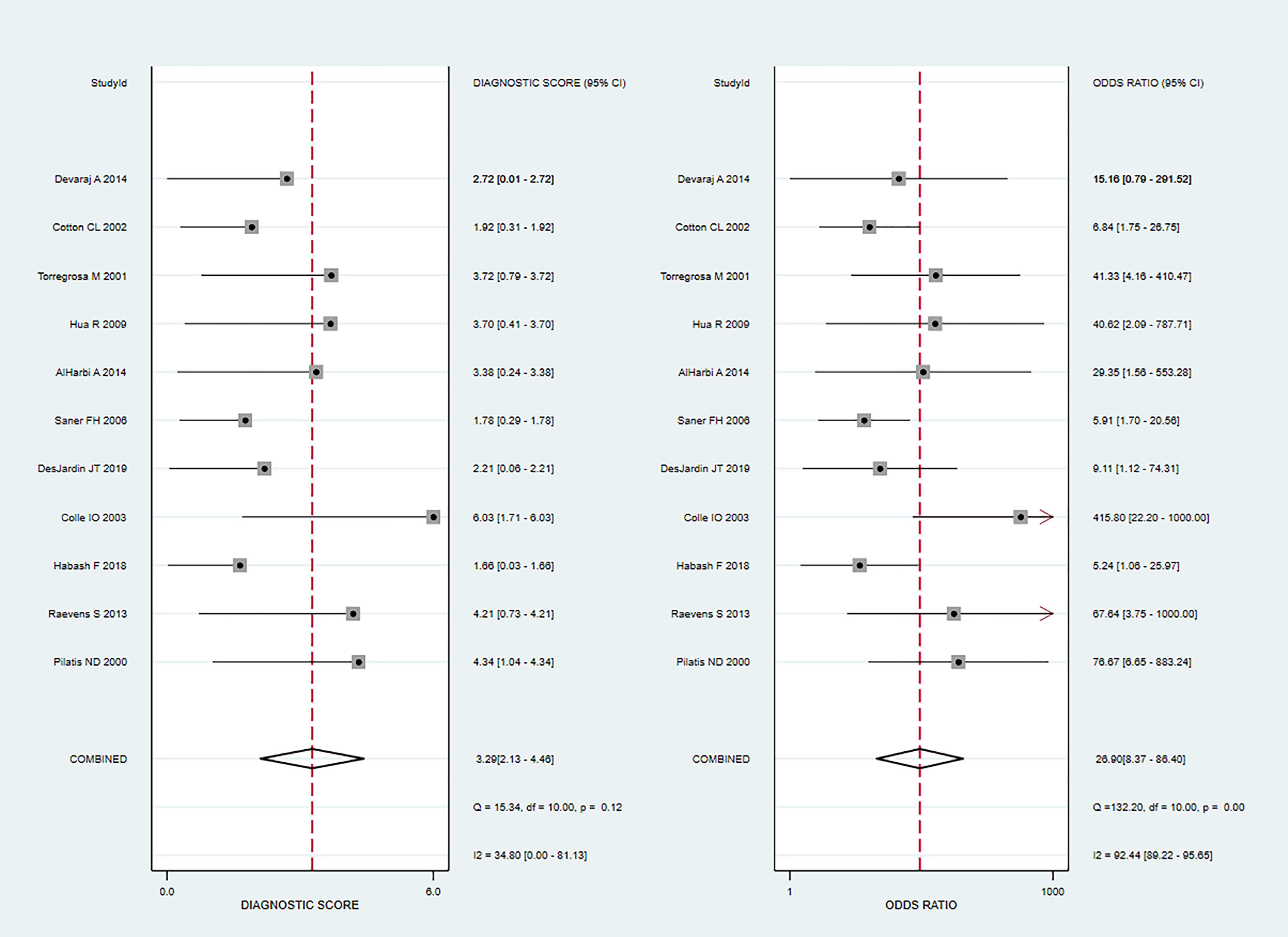

Supplement: Figure S3 [file peerj-08-9243-s003.png]
