# Supplementary material for: Role of echocardiography in screening for portopulmonary hypertension in liver transplant candidates: a meta-analysis
Source: PeerJ. 2020 May 27;8:e9243. doi: 10.7717/peerj.9243 (PMC7261122; doi:10.7717/peerj.9243)
Supplement: File S1 — We extracted the relevant data in the paper for statistical analysis, and concluded that echocardiography plays an important role in the screening of POPH. [file peerj-08-9243-s004.docx]

| **First author** | **TP** | **FP** | **FN** | **TN** |
| --- | --- | --- | --- | --- |
| Pilatis ND | 5 | 1 | 3 | 46 |
| Raevens S | 7 | 26 | 0 | 119 |
| Habash F | 10 | 3 | 7 | 11 |
| Colle IO | 10 | 7 | 0 | 148 |
| DesJardin JT | 10 | 45 | 1 | 41 |
| Saner FH | 9 | 14 | 5 | 46 |
| AlHarbi A | 4 | 57 | 0 | 187 |
| Hua R | 4 | 18 | 0 | 83 |
| Torregrosa M | 4 | 9 | 1 | 93 |
| Cotton CL | 6 | 10 | 5 | 57 |
| Devaraj A | 5 | 18 | 0 | 25 |

Raw data
