# Supplementary material for: Role of echocardiography in screening for portopulmonary hypertension in liver transplant candidates: a meta-analysis
Source: PeerJ. 2020 May 27;8:e9243. doi: 10.7717/peerj.9243 (PMC7261122; doi:10.7717/peerj.9243)
Supplement: Supplemental Information 1 [file peerj-08-9243-s005.doc]

| **Section/topic** | **#** | **Checklist item** | **Reported on page #** |
| --- | --- | --- | --- |
| **TITLE** | | |  |
| Title | 1 | Role of echocardiography in screening for portopulmonary hypertension in liver transplant candidates: A meta-analysis | Page1 paragraph1 sentence1 |
| **ABSTRACT** | | |  |
| Structured summary | 2 | Objectives. To demonstrate the screening value of echocardiography for portopulmonary hypertension (POPH) in liver transplant candidates.  Design. Systematic review and meta-analysis  Background. POPH is a complication of end-stage liver disease that adversely affects the outcome of orthotopic liver transplant. There are no specific symptoms in the early stage of POPH. POPH reduce the survival rate of patients with end-stage liver disease specially if they are not diagnosed. Therefore, early detection may improve prognosis. The objective of this study is to explore the screening value of echocardiography on liver transplant candidates for screening of POPH compared to right heart catheterization (RHC).  Method. PubMed, EMBASE and the Cochrane Library were searched by two independent reviewers for potentially eligible studies published up to 30 June 2019 to retrieve data based on per-patient analysis. STATA, Meta-DiSc, and RevMan were applied to perform this meta-analysis.  Results. Our search yielded 1576 studies, of which 11 satisfied the inclusion criteria. The pooled sensitivity, specificity, positive likelihood ratio (PLR), negative likelihood ratio (NLR) and area under the summary receiver operating characteristic (SROC) curve (AUC) of echocardiography for POPH were 0.85 (95% CI, 0.65-0.94), 0.83 (95% CI, 0.73-0.90), 4.99 (95% CI, 3.03-8.21), 0.19 (95% CI, 0.07-0.46), and 0.91 (95% CI, 0.88-0.93), respectively. Deeks' funnel plot did not indicate the existence of publication bias (P= 0.66).  Conclusions. Echocardiography, a noninvasive modality, provides superior screening for POPH, but the diagnosis of POPH still requires RHC.  PROSPERO registration number  CRD42019144589 | Page2 Page3 paragraph2 and paragraph 3 |
| **INTRODUCTION** | | |  |
| Rationale | 3 | This is the first meta-analysis to assess the value of echocardiography as a screening tool for POPH.  The methodological quality of each study was assessed using Quality Assessment of Diagnostic Accuracy Studies 2 (QUADAS-2). | Page3 paragraph5 and paragraph6 |
| Objectives | 4 | We performed this meta-analysis, which may complement existing studies, to evaluate the accuracy of echocardiography compared to RHC as a screening method in liver transplantation patients. | Page4 paragraph 2 |
| **METHODS** | | |  |
| Protocol and registration | 5 | This meta-analysis was registered in PROSPERO, and the registration number was CRD42019144589. | Page5 paragraph1 sentence1 |
| Eligibility criteria |  | (1) patients included in the studies were liver transplantation candidates who underwent echocardiography and RHC before liver transplantation; (2) the results of RHC served as a reference standard for diagnosis and severity; (3) a certain cut-off values for echocardiography was adopted to screen POPH patients; (4) effectives tricuspid regurgitation was demonstrated by echocardiography and pulmonary artery pressure was estimated; and (5) the extracted data were available to calculate true positive, false positive, false negative and true positive values. | Page5 paragraph2 |
| Information sources | 7 | PubMed, EMBASE and the Cochrane Library were searched by two independent reviewers (Xin Yin and Yueming Shao) for potentially eligible studies published up to 30 June 2019. | Page5 paragraph1 sentence2 |
| Search | 8 | The search strategy was as follows: (“Portopulmonary hypertension” or “porto pulmonary hypertension” or “POPH” or “PPH” or “PPHTN”) and (“echocardiography”). The “All fields” category was used for search. | Page5 paragraph1 sentence4 |
| Study selection | 9 | The studies were required to meet the following criteria: (1) patients included in the studies were liver transplantation candidates who underwent echocardiography and RHC before liver transplantation; (2) the results of RHC served as a reference standard for diagnosis and severity; (3) a certain cut-off values for echocardiography was adopted to screen POPH patients. (4) effectives tricuspid regurgitation was demonstrated by echocardiography and pulmonary artery pressure was estimated; and (5) the extracted data were available to calculate true positive, false positive, false negative and true positive values. The exclusion criteria of this study were as follows: (1) non-English articles; (2) case reports, conference abstracts, reviews, editorial materials, letters, and comments; and (3) studies involving the individuals. | Page5 paragraph2 sentence2-3  and Page6 paragraph1 sentence1 |
| Data collection process | 10 | Two independent reviewers (Xin Yin and Hui Gao) extracted the following information: first author, year, sample size, mean/median age, the number of POPH/non-POPH patients, cut-off value, false negative, false positive, true negative, true positive. Disagreement was solved by discussion and if necessary, a third reviewer (Tingting Qin) was involved to reach a consensus. Quality assessment was assessed by two independent researchers (Xiaoyu Wen and Chen Yang) using Quality Assessment of Diagnostic Accuracy Studies 2 (QUADAS-2). | Page6 paragraph 2 |
| Data items | 11 | Two independent reviewers (Xin Yin and Hui Gao) extracted the following information: first author, year, sample size, mean/median age, the number of POPH/non-POPH patients, cut-off value, false negative, false positive, true negative, true positive. Disagreement was solved by discussion and if necessary, a third reviewer (Tingting Qin) was involved to reach a consensus. | Page6 paragraph 2 sentence 1-2 |
| Risk of bias in individual studies | 12 | The P-value of the Spearman correlation coefficient was used to measure the threshold effect. A P-value greater than 0.05 indicated that there was no threshold effect and that further exploration into whether heterogeneity was caused by a non-threshold effect was needed. | Page6 paragraph3 sentence 2-3 |
| Summary measures | 13 | The combined sensitivity, specificity, positive likelihood ratio (PLR), negative likelihood ratio (NLR), and their 95% confidence intervals (CIs) were calculated and graphically shown using forest plots. | Page6 paragraph3 sentence 5 |
| Synthesis of results | 14 | The heterogeneity was evaluated by the value of the I-square statistic using the “midas” command based on a bivariate model of a hierarchical receiver operating characteristic (HSROC). The combined sensitivity, specificity, positive likelihood ratio (PLR), negative likelihood ratio (NLR), and their 95% confidence intervals (CIs) were calculated and graphically shown using forest plots. A summary receiver characteristic curve (SROC) was applied to assess the screening accuracy of echocardiography, and the AUC was computed; the higher the AUC, the higher the screening value was. | Page6 paragraph3 sentence 4-6 |

| **Section/topic** | **#** | **Checklist item** | **Reported on page #** |
| --- | --- | --- | --- |
| Risk of bias across studies | 15 | Deeks’ funnel plot asymmetry test was applied to assess publication bias. | Page6 paragraph3 sentence7 |
| Additional analyses | 16 | Additionally, we combined the Pearson's correlation coefficient of echocardiography and RHC in liver transplantation individuals using the “metacor” package of R software (version 3.5.3). | Page7 paragraph1 sentence 2 |
| **RESULTS** | | |  |
| Study selection | 17 | A total of 1089 articles were retrieved by electronic search after duplicates were excluded. Based on the inclusion and exclusion criteria, 11 full-text articles were ultimately included in the meta-analysis. A PRISMA flow diagram of the retrieved studies is shown in Figure 1. | Page7  Paragraph2 |
| Study characteristics | 18 | For each study, present characteristics for which data were extracted (e.g., study size, PICOS, follow-up period) and provide the citations. | Table 1 |
| Risk of bias within studies | 19 | Present data on risk of bias of each study and, if available, any outcome level assessment (see item 12). | Figure S1 |
| Results of individual studies | 20 | For all outcomes considered (benefits or harms), present, for each study: (a) simple summary data for each intervention group (b) effect estimates and confidence intervals, ideally with a forest plot. | Figure 2 |
| Synthesis of results | 21 | Present results of each meta-analysis done, including confidence intervals and measures of consistency. | Figure 2 |
| Risk of bias across studies | 22 | Present results of any assessment of risk of bias across studies (see Item 15). | Figure S1 |
| Additional analysis | 23 | Give results of additional analyses, if done (e.g., sensitivity or subgroup analyses, meta-regression [see Item 16]). | None |
| **DISCUSSION** | | |  |
| Summary of evidence | 24 | Our study confirms that the screening accuracy of echocardiography for POPH is clinically acceptable, showing a sensitivity of 0.85 (95% CI, 0.65-0.94), specificity of 0.83 (95% CI, 0.73-0.90), and area under the SROC curve of 0.91 (95% CI, 0.88-0.93). Two meta-analyses were previously conducted to evaluate the diagnostic value of echocardiography in pulmonary hypertension (PH). A meta-analysis by de Surinder showed that the estimated sensitivities and specificity of echocardiography for patients with PH were 83% and 72%Error: Reference source not found, respectively. Mohammed et alError: Reference source not found conducted a meta-analysis of 9 studies among patients with PH and found that echocardiography had a sensitivity of 88% and a specificity of 56% for PH patients. However, these two studies were meta-analyses based on echocardiography as a diagnostic tool for patients with PH. Our article looked at a special group of patients with PH and is a comprehensive study of reports up to 31 June 2019. Our results showed that echocardiography had high sensitivity and specificity for detecting POPH in liver transplantation candidates. | Page10 paragraph2  sentence5-11 |
| Limitations | 25 | There were several implicit limitations in our meta-analysis. First, we included only studies published in PubMed, EMBASE and the Cochrane Library, and we excluded abstracts, letters to the editor and articles written in languages other than English. This may have led to publication bias. Second, the time interval between echocardiography and RHC was different. The longer the period between echocardiography and RHC, the higher the chance that the hemodynamic status of patients will change. Third, 1 of the 11 articles estimated right atrial pressure based on a fixed value of 10 mmHg. In other studies, right atrial pressure was estimated using the inferior vena cava diameter. The use of the jugular venous pressure for clinical estimates does not allow reliable measure of right atrial pressure and is less satisfactory than using a fixed value of 14 mm Hg to predict pulmonary artery pressure. Therefore, we think that the use of a fixed value of 10 mmHg has little effect on the value of pulmonary artery systolic pressure. All of the above factors increase the heterogeneity of the studies. In our study, the heterogeneity was high, but the generality of this conclusion may be affected by the absence of grouping basis for a subgroup analysis and the inability to conduct further related subgroup analysis. Consequently, our conclusions need to be interpreted with caution. | Page10 paragraph2 |
| Conclusions | 26 | In summary, echocardiography is a highly sensitive tool for noninvasive screening of POPH. However, if the echocardiography results are abnormal, RHC should be performed to confirm the diagnosis. Our study provides a basis for echocardiography as a POPH screening tool. Moreover, further larger prospective studies are recommended to verify the comprehensive effectiveness of echocardiography as a noninvasive means for detecting patients with POPH. | Page11 paragraph2 |
| **FUNDING** | | |  |
| Funding | 27 | Describe sources of funding for the systematic review and other support (e.g., supply of data); role of funders for the systematic review. | None |

*From:*  Moher D, Liberati A, Tetzlaff J, Altman DG, The PRISMA Group (2009). Preferred Reporting Items for Systematic Reviews and Meta-Analyses: The PRISMA Statement. PLoS Med 6(7): e1000097. doi:10.1371/journal.pmed1000097

For more information, visit: **www.prisma-statement.org**.

Page 2 of 2
