# Supplementary material for: Role of echocardiography in screening for portopulmonary hypertension in liver transplant candidates: a meta-analysis
Source: PeerJ. 2020 May 27;8:e9243. doi: 10.7717/peerj.9243 (PMC7261122; doi:10.7717/peerj.9243)
Supplement: Supplemental Information 2 [file peerj-08-9243-s006.docx]

1. The rationale for conducting the meta-analysis

This study is the first meta-analysis that summarizes the diagnostic accuracy of echocardiography for portopulmonary hypertension (POPH).

2.The contribution that the meta-analysis makes to knowledge in light of previously published related reports, including other meta-analyses and systematic reviews.

There have been two meta-analyses to evaluate the diagnostic value of echocardiography in pulmonary hypertension (PH). A meta-analysis by de Surinder showed that echocardiography estimated sensitivities and specificity for patients with PH were 83% and 72%, respectively. Mohammed et al conducted a meta-analysis including 9 studies among patients with PH and found that echocardiography had a sensitivity of 88% and a specificity of 56% for PH patients. This study includes POPH, a special group of patients with pulmonary hypertension, and it contains a comprehensive study up to 31 June 2019.
